# TV-based assistive integrated service to support European adults living with mild dementia or mild cognitive impairment (TV-AssistDem): study protocol for a multicentre randomized controlled trial

**DOI:** 10.1186/s12877-019-1267-z

**Published:** 2019-09-06

**Authors:** Jessica Marian Goodman-Casanova, José Guzmán-Parra, Gloria Guerrero, Elisa Vera, Pilar Barnestein-Fonseca, Gabriella Cortellessa, Francesca Fracasso, Alessandro Umbrico, Amedeo Cesta, Diana Toma, Flavia Boghiu, Rodolphe Dewarrat, Valentina Triantafyllidou, Elena Tamburini, Pietro Dionisio, Fermín Mayoral

**Affiliations:** 1 Instituto de Investigación Biomédica de Málaga – Fundación Pública Andaluza para la Investigación de Málaga de Biomedicina y Salud (IBIMA-FIMABIS), Málaga, Spain; 2 CNR–Italian National Research Council, ISTC, Rome, Italy; 3 S.C. INGRIJIRI LA DOMICILIU S.R.L., Brasov, Romania; 4 Institut multidisciplinaire de science de donnees (IMSD), Les Reussilles, Switzerland; 5 MEDEA, Massa, Italy

**Keywords:** Ambient assisted living, Assistive technology, Telemedicine, Remote consultations, Dementia, Person with dementia, Informal caregivers, Quality of life

## Abstract

**Background:**

Mild cognitive impairment and mild dementia progressively compromise the ability of people to live independently and can have a negative impact on their quality of life. Within the current European Active and Assisted Living programme (AAL), project TV-AssistDem has been developed to deliver a TV-based platform service to support patients with mild cognitive impairment or mild dementia and provide relief to their caregivers. The application is intended to be used daily at home, mainly by the participants themselves, with the help of their informal caregivers. The aim of this study is to evaluate the effectiveness of TV-AssistDem to improve quality of life in people with mild cognitive impairment or mild dementia.

**Methods:**

This is a 12-month European multicentre randomized controlled trial which will be performed in two countries: Spain and Romania. Two hundred and forty older adults will be recruited using identical inclusion/exclusion criteria. The primary outcome will be the change from baseline of TV-AssistDem on patient quality of life at 12 months. The secondary outcomes will be the changes from baseline of: 1) informal caregiver quality of life, 2) informal caregiver burden, 3) patient treatment adherence, 4) patient treatment compliance, 5) patient functional status, and 6) healthcare cost-effectiveness at 12 months. Patients in the intervention group will have access to an interactive platform which offers remote assistive services through a device connected to the television. The core services of the platform are: 1) Calendar and reminders, 2) Health monitoring and data transmission to a health server and 3) Videoconference; service-oriented applications are: 4) Cognitive stimulation; 5) Reminiscences; and 6) Patient and caregiver healthcare education. The analysis will be made following an intention-to-treat procedure. Linear and Generalized Mixed Model analysis will be performed.

**Discussion:**

We hypothesize that the regular use of TV-AssistDem will result in an improvement in patient quality of life. The uniqueness of this home TV-based intervention lies on its widespread accessibility and its integrative approach to quality of life in people with mild cognitive impairment or mild dementia and their informal caregivers. However, several anticipated challenges will need to be faced: poor engagement and connectivity problems.

**Trial registration:**

ClinicalTrials.gov Identifier NCT03653234, Date of registration: 31 August 2018.

## Background

Population ageing within Europe has major social and economic consequences. The burden of non-communicable disease and disability increases with age, exerting pressures on health services and support systems for older people. One of the most devastating conditions that predominantly affects older people is dementia [1]. Dementia is an umbrella term describing a set of symptoms that occur when various diseases or conditions affect the brain, being the most common cause of dementia, Alzheimer’s disease. The symptoms that comprise dementia can vary greatly, with people often experiencing memory loss but also problems in communication and attention, anxiety and depression [2]. Mild cognitive impairment represents an intermediate state between normal cognitive changes when aging and the earliest clinical manifestations of dementia. It is a syndrome where decline in cognition in a person is greater than the level expected for his/her age and education level, but which does not interfere notably with activities of daily life. The distinction between dementia and mild cognitive impairment can be quite subtle [3]. Treatment options currently remain centered on symptomatic treatment. The main drugs prescribed for people with mild to moderate dementia are cholinesterase inhibitors. Anti-dementia drugs have not yet met the scientific community’s expectations.

There are currently about 10.5 million people in Europe with dementia, costing around 275 billion euros annually. Given that this number is expected to rise to 13.4 million by 2030, the challenge of dementia is likely to remain formidable [4]. Healthcare policies are therefore focused on extending the ability of older people to continue to live independently. This entails maintaining the quality of life (QoL) of people with mild cognitive impairment or mild dementia (PMCI/MD), as well as, working to reduce the costs of their care.

Living with dementia in the early stages can be burdensome for both those affected as well as their caregivers [5, 6]. Caring for PMCI/MD can compromise the informal caregiver’s own well-being and health due to a feeling of being overloaded in the caregiving role [7]. The well-being of the informal caregivers themselves and providing them with support is therefore essential [8].

The importance of including QoL as an outcome measure in dementia trials has been identified by the World Federation of Biological Psychiatry’s Old Age Taskforce [9]. QoL is multifactorial; consequently, in addition to “an individual’s perception of their position in life in the context of the culture and value system in which they live and in relation to their goals, expectations and standards and concerns.” [10], it also includes physical and mental health, social relations and participation in activities. Therefore, it cannot be determined solely by changes in cognition or other symptoms.

Systematic reviews focused on the effectiveness of interventions in QoL of PMCI/MD conclude that while pharmacological interventions seek changes in symptoms as a main outcome and have not been found to improve QoL, non-pharmacological interventions aimed at PMCI/MD living in their home and their caregivers have proven QoL improvement [9].

Information and Communications Technology (ICTs) studies focus on the use of assistive technologies to improve QoL in elderly people in general [11]. These technologies are computers, sensors, telemedicine, mobile applications of medication management, etc. The age-related digital divide and cognitive, sensorial and psychomotor impairment determine the use of certain ICTs in older people, such as tablets (e.g. losing the tablet or not being able to visually see the tablet’s screen). Television sets however, are standard devices with an extensive home penetration, being TV-based interventions more suitable for this population.

Three ICTs studies were identified which show QoL improvement in PMCI/MD and their caregivers. A recent clinical review on digital technologies used to monitor cognition, identified a range of digital systems, such as smartphones, smartwatches and smart homes, to assess and assist elderly demented, prodromal and preclinical populations. It highlights the need for additional research that validates the efficacy, utility and cost-effectiveness of these systems in patient populations [12]. However, there are few large evidence based studies on the effectiveness of these techs to improve QoL in PMCI/MD. These studies have small sample sizes, do not include QoL as a main outcome measure, or do not include valid QoL scales. Therefore, randomized controlled clinical trials with large samples are needed.

Since QoL also includes physical and mental health, follow-up of agreed recommendations from a health care provider in terms of medication and Activities of Daily Living (ADLs) will be crucial [13]. Patient adherence to prescribed medication is probably the most important therapeutic factor and a determinant of medication effectiveness and safety [14, 15]. Scientific literature reveals poor cognitive function as a risk factor of medication non-adherence [16]. Often, a patient’s ability to manage his or her medication is decisive regarding whether a patient has mild cognitive impairment or dementia, which is why assessing medication adherence is crucial [16].

Group Structured Cognitive Stimulation Therapy (CST) is recommended by the NICE-SCIE Guideline on Supporting people with Dementia and their caregivers [17]. Home-based CST is an opportunity for people with health/mobility problems or who dislike group therapy and increases accessibility for local areas which do not offer group therapy [18].

There has been a raise in the development of ICTs to improve or to enhance different domains to keep the independence or to support PMCI/MD like: reminders, cognitive training, geolocation devices, but all of them have focused in monitoring cognition with limited data on patient QoL.

The aim of this study is to deliver a TV-based platform service which will improve quality of life of people with mild cognitive impairment or mild dementia. Additionally, this technological solution seeks to improve caregiver quality of life and reduce caregiver burden, increase patient treatment adherence and compliance, reduce patient functional decline, and lower healthcare costs.

## Methods

### Aims

We refer to our TV-based Assistive Integrated Service intervention as TV-AssistDem. The main objective of this project is:

1. To evaluate the effectiveness of TV-AssistDem on quality of life among people with mild cognitive impairment or mild dementia after 12 months follow-up in comparison to controls. We hypothesize that the use of TV-AssistDem over 12 months can result in an improvement in quality of life when compared to a matched control group of patients not using TV-AssistDem.

The secondary objectives of this project are:

1. To evaluate the impact of TV-AssistDem on caregiver quality of life after 12 months of follow-up. We hypothesize that quality of life will be significantly improved in caregivers after the intervention when compared to matched controls over 12 months.
2. To evaluate the impact of TV-AssistDem on caregiver burden after 12 months of follow-up. We hypothesize that burden will be significantly reduced in caregivers after the intervention when compared to matched controls over 12 months.
3. To evaluate the impact of TV-AssistDem on patient treatment adherence after 12 months of follow-up. We hypothesize that treatment adherence will be significantly increased in patients in the intervention group when compared to matched controls over 12 months.
4. To evaluate the impact of TV-AssistDem on patient treatment compliance after 12 months of follow-up. We hypothesize that treatment compliance will be significantly increased in patients in the intervention group when compared to matched controls over 12 months.
5. To evaluate the impact of TV-AssistDem on functional status after 12 months of follow-up. We hypothesize that functional decline will be significantly reduced in patients in the intervention group when compared to matched controls over 12 months.
6. To test the economic and financial impacts of using the TV-AssistDem after 12 months of follow-up. We hypothesize that after accounting for the estimated per patient costs of our intervention, patients in the intervention group will have significantly lower healthcare costs after 12 months follow-up when compared to matched controls over 12 months.

### Study design

This study is a multicentre randomised controlled trial and will be performed in two European countries: Spain and Romania. The participant centres are: the Spanish Health Research Institute of Malaga (IBIMA) and the Romanian NGO Home Care Association (Îngrijiri La Domiciliu).

### Setting

Patients will be identified from people with self-perceived cognitive impairment or caregiver’s perception of cognitive impairment that has been present for more than 6 months and who meet all the study eligibility criteria. The patients can be under primary care services, as well as, secondary care services, such as those who are being followed up in memory clinics, outpatient clinics, day hospitals or other components of specialist mental health, geriatric medicine and neurology services. The clinicians involved in identifying the patients will be physicians (primary care, psychiatry, neurology, and geriatrics), neuropsychologists and dementia and mental health nurses.

The identification process by the clinician will consist of screening, using information gathered from medical notes, clinical records and/or clinical consultations, for initial eligibility based on inclusion criteria. Patients will receive a brief explanation by their clinician. A member of the research team will contact the patient to see if they are willing to participate in the study, and if so, an appointment at their referral centre will be scheduled. Their primary caregiver must attend this visit.

At this first visit, the researcher will determine the patient’s eligibility and will explain the study in detail and answer any questions the patient or caregiver may have. After doing so, the patient’s ability to consent will be assessed following the Assessment of capacity to consent which includes the 5 fundamental principles of the Mental Capacity Act. In case of cognitive impairment or dementia, patients will be asked five questions regarding the information received as part of the decision-making process. Proxy consent of a legal representative or the caregiver will be required when the patient is not found capable. Both the patient and caregiver will give consent regarding their participation in the study. Once given consent, the patient will be randomized into the intervention or control group. Follow-up will begin after the randomization and will be determined by the group allocation. Within one to two weeks after being included in the study, patients allocated in the intervention group will be scheduled a visit for TV-AssistDem installation and training.

Patients will be assessed for 12 months. After the baseline visit in which all the variables of the study will be collected, follow-up visits will be carried out every 6 months in both the control group and the intervention group. In these follow-up visits, researchers will assess the QoL of the PMCI/MD, adherence and compliance to prescribed medication, functional status, and service utilization. Researchers will also evaluate the QoL of caregivers and their burden. In addition, in the intervention group, TV-AssistDem user-behaviour data will be collected and usability will be evaluated.

Recruitment is scheduled to start in March 2019 and end in May 2019. Follow-up assessments will be ongoing and will be completed mid-2020.

### Participants

Two hundred forty participant dyads in two European countries (Spain and Romania). The participant dyads will comprise the PMCI/MD and their informal caregivers, defined as the person who is unwaged for this role and who spends the most time with the patient and whom the patient declares that he/she is his/her informal caregiver, for care or support of care, and who does not take part in a formal network of organized care.

A patient will be eligible for inclusion in this trial only if all following criteria apply:

- Patients are over sixty years of age.
- Self-perceived cognitive impairment or caregiver’s perception of cognitive impairment that has been present for more than 6 months.
- Patients score 23–27 points on Mini-Mental State Examination (MMSE).
- Patients live independently.
- Patients have an informal caregiver.
- Patients who take pharmacological treatment for chronic conditions during study period and are in charge of their own medication.
- The place where patients live has enough wireless or phone network connectivity to enable them to use TV-AssistDem platform on a daily basis.
- Patients agree to be part of the study by giving signed written consent.

A patient will not be eligible for inclusion in this trial if any of the following criteria apply:

- Patients score above 11 on the Geriatric Depression Scale (GDS-15).
- Patients have a terminal illness with less than 3 years expected survival.
- Patients have specific conditions reducing their physical ability to use the application to a point that makes their participation in the project impossible (the nature of the conditions will be recorded in such cases).

### Outcome measures

The primary outcome measure is the change in patient quality of life measured with the Quality of Life-Alzheimer’s Disease Scale (QoL-AD) [19–22]. Additional assessments will be used for secondary outcomes: caregiver quality of life and burden, patient treatment adherence and compliance, patient functional status and service utilization. TV-Assist Dem usability and other covariables will also be collected.

Patients will be assessed at baseline and at 6 months and 12 months after the beginning of the intervention. The assessments will be conducted under standardized conditions at the study centres.

#### Primary outcome

##### Patient quality of life

QoL-AD is an instrument specifically designed to measure QoL in PMCI/MD from the perspective of both the patient and the informal caregiver. It is a 13-item measure, which includes assessments of the person’s relationships with friends and family, financial situation, physical condition, mood, memory, and an overall assessment of life quality. Response are 4-point multiple choice options (1 = poor, 2 = fair, 3 = good, 4 = excellent). Scale scores range from 13 to 52, with higher scores indicating greater quality of life. PMCI/MD may lose cognitive function during the study to the extent that they are unable to complete the assessments themselves. For this reason, informal caregivers will also complete the QoL-AD, in parallel and on behalf of the PMCI/MD, from the start of the study. QoL-AD will be assessed via interview with the patient and via self-completion with caregivers at baseline and then at 6 and 12 months.

#### Secondary outcomes

##### Caregiver quality of life

EuroQoL-5D-5 L [23, 24] is a standardized generic instrument consisting of a descriptive system and a visual analogue scale (VAS).

The descriptive system comprises 5 dimensions covering mobility, self-care, usual activities, pain/discomfort and anxiety/depression. Each dimension has 5 levels: no problems, slight problems, moderate problems, severe problems and extreme problems. A 1-digit number expresses the level selected for that dimension. The digits can be combined into a 5-digit number that describes the patient’s health state.

The VAS records the patient’s self-rated health on a vertical scale, where the endpoints are ‘The best health you can imagine’ and ‘The worst health you can imagine’.

EuroQoL-5D-5 L has been shown to correlate well with QoL-AD, indicating that using both measures side-by-side is compatible [19]. Both will be completed by the patient and caregiver at baseline and then at 6 and 12 months.

##### Caregiver burden

The Zarit Burden Interview (ZBI-12) [25, 26] will be used to evaluate the informal caregivers’ burden. The ZBI-12 is a 12-item scale with each answer chosen from a 5-point Likert scale (Nearly always = 4, Quite frequently = 3, Sometimes = 2, Rarely = 1, Never = 0). It is a shortened version of the original scale, was developed specifically for informal caregivers of PMCI/MD and covers issues such as caregiver stress and the degree to which caring is affecting their health and social life. Total score ranges 0 to 48 (0–10 = no to mild burden, 10–20 = mild to moderate burden > 20 = high burden). It will be administered via interview with the informal caregiver at baseline and then at 6 and 12 months.

##### Treatment adherence

The dose/pill count is the number of pills or doses taken divided by the prescribed ones, multiplied by 100 (expressed as a percentage) [27, 28]. According to Haynes et al. recommendations [32], a good adherence is considered when the result of counting is between 80% (a 20 % of doses/pills missed) and 110% (the patient takes 10 % more doses/pills) of dose/pill prescribed [29].

The documented prescription for medications will be compared with the number of pills taken (or pill equivalents) 30 days before assessment day. Dyads will be asked to bring to the visit any documentation related with their prescription history, all the pills and empty medication packaging from the previous 30 days, so that the researcher can undertake the medication count. Drugs considered to evaluate adherence will be anti-dementia drugs and other chronic diseases treatment that influence the evolution of dementia (e.g. drugs for cardiovascular diseases, antidiabetics, anticoagulants, antihypertensive and antidepressants). This assessment will be undertaken at baseline and then at 6 and 12 months.

##### Treatment compliance

The Medication Assessment Questionnaire test was originally developed to assess medication compliance in patients with hypertension [30]. Since the test was introduced it has been used in the assessment of therapeutic compliance in different diseases and has been validated for Spanish population [31]. It consists of 4 contrast questions with dichotomous yes/no responses, reflecting the patient’s behaviour regarding compliance. They are intended to assess whether the patient adopts correct attitudes regarding treatment for their disease; it is assumed that if the attitudes are incorrect the patient is non-compliant. It will be administered at baseline and then at 6 and 12 months.

##### Functional status

Lawton Instrumental Activities of Daily Living Scale (IADL) [32] scale will be used to evaluate functional status in the PMCI/MD. The IADL is an interview format scale of 8-items covering instrumental activities, which are required for independent living: ability to use telephone, shopping, food preparation, housekeeping, laundry, mode of transportation, responsibility for own medications and ability to handle finances. Because these instrumental functions are usually lost before more basic ADLs functions (such as bathing, eating, and using the toilet), assessment of IADL may identify incipient decline in older people who might otherwise appear capable and healthy. The score ranges from 0 (low function, dependent) to 8 (high function, independent) for women and 0 through 5 for men to avoid potential gender bias. IADL will be administered via interview to the PMCI/MD and their caregiver at baseline, and then at 6 and 12 months.

##### Service utilization

The Client Service Receipt Inventory (CSRI) [33, 34] scale will be used to evaluate the service utilization. This scale is an internationally used method for gathering data on service utilization and other domains relevant for economic analysis of mental health care. It has five sections: background client information, accommodation and living situation, employment history, earnings and benefits, a record of services usually used and information about informal caregiver support. The sections assessed will be consultations, admissions and visits in the last year, grouped into subsections according to hospital, specialist, primary or home care. Treatment related to hospital admissions or illness exacerbation will also be assessed. It’s adaptability ensures it is compatible with the research aims, context, participants’ likely circumstances, and the quantity and precision of information required. The CSRI will be administered via interview with PMCI/MD at baseline and at 12 months.

##### Screening tools

The Mini-Mental State Examination [35] will be used to assess the cognitive function of the PMCI/MD and determine their eligibility for participation. There is a great heterogeneity among studies regarding the cutoff scores for dementia and mild cognitive impairment. The most common scores are ranging 23 to 27, however, a person’s age and educational level determines the accuracy of this cut-point.

The Short Form of the Geriatric Depression Scale [36] will be used to rule out depressive pseudo-dementia which is considered a reversible form of cognitive deficit. PMCI/MD scoring over 11 will not be eligible for participation.

##### Covariables

Covariables will be Sociodemographic data (marital status, level of education, living arrangements and hobbies) and Medical history (smoking habit, disabilities, anthropometric data, cognitive impairment data, comorbidities and need for vital sign monitoring).

##### User-behaviour

A user-behaviour analysis will be performed to assess how users interact with the platform and how their behaviour affects its effectiveness. We will assess and analyze the frequency of access to the platform itself and to each functionality and the length of each interaction. These data will be correlated with users’ reminder schedules to explore differences between proactive and reactive use of the platform and will be followed up over time to understand how increasing familiarity with the platform affects user-behaviour. Additionally, interviews will be scheduled with 5% of randomly selected users after 12 months of using the platform to assess their user experience.

##### Usability evaluation

The System Usability Scale (SUS) [37, 38] will be used to address how well patients learn and use TV-AssistDem. It is a simple, 10-item scale giving a global view of subjective usability. The respondent indicates the degree of agreement or disagreement with each statement on a 5-point Likert scale with a score range of 0 to 100. The SUS scale will be used after the patients have had an opportunity to use TV-AssistDem before any debriefing or discussion takes place at 6 and 12 months.

The collected variables and data collection time are presented in Tables 1 and 2.

**Table 1:** Patient’s data collection table

Assessment	Outcome variable	Screening	Baseline	6 months	12 months
Informed Consent	NA	X			
Capacity to Consent	NA	X			
Mini-Mental State Examination	NA	X		X	X
Geriatric Depression Scale	NA	X		X	X
Sociodemographic data	NA		X	X	X
Medical history	NA		X	X	X
Quality of Life-Alzheimer’s Disease Scale	Primary		X	X	X
EuroQoL-5D-5 L	Secondary		X	X	X
Medication Assessment Questionnaire	Secondary		X	X	X
Lawton Instrumental Activities of Daily Living Scale	Secondary		X	X	X
Client Service Receipt Inventory	Secondary		X		X
System Usability Scale	NA			X	X

*NA* = non-applicable

**Table 2:** Caregiver’s data collection table

Assessment	Outcome variable	Screening	Baseline	6 months	12 months
Informed Consent	NA	X			
Caregivers data	NA	X			
Quality of Life-Alzheimer’s Disease Scale	Primary		X	X	X
EuroQoL-5D-5 L	Secondary		X	X	X
Zarit Burden Interview	Secondary		X	X	X
Lawton Instrumental Activities of Daily Living Scale	Secondary		X	X	X

*NA* = non-applicable

### Intervention and control group

#### Intervention group

Intervention will consist in the use of TV-AssistDem: an interactive platform which offers remote assistive services through a device connected to the television. Patients and caregivers have been actively involved in designing the 6 main functionalities of this innovative technology. Adapting the functionalities and content to best fit PMCI/MD and their caregivers has been crucial. Focus groups have been carried out and valuable indications have been gathered for the platform development. The TV-AssistDem prototype will be validated through a pre-pilot feasibility study which will include a User acceptance report.

**Fig. 1:**
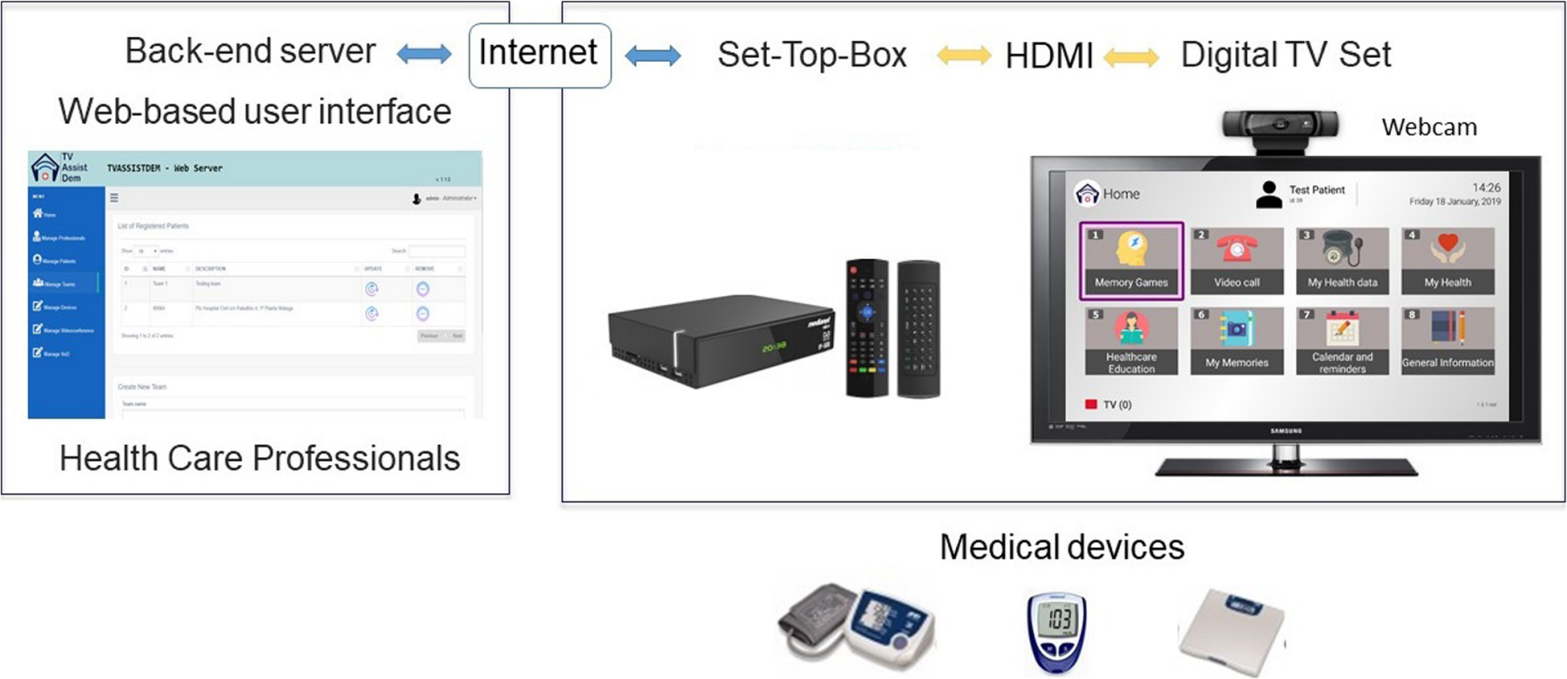
TV-AssistDem system

The product consists of an Android software and hardware TV (a Digital TV Set Top Box with Android technology) designed to provide tele-care services at home using the TV. The system consists of (Fig. 1):

- A Set Top Box which allows end-users to enjoy TV-AssistDem services. It’s based on ARM chipsets with Android operating system and a digital TV tuner (optional, including HDMI pass-through) compatible with DVB-T/T2 (European version).
- A webcam which enables the videoconference service.
- A centralized back-end server which is responsible for managing and storing information about patients and providing the basic functionalities needed for TV-AssistDem services.
- A web-based user interface which allows researchers to manage general information, events and appointments concerning the associated primary patients.

**Fig. 2:**
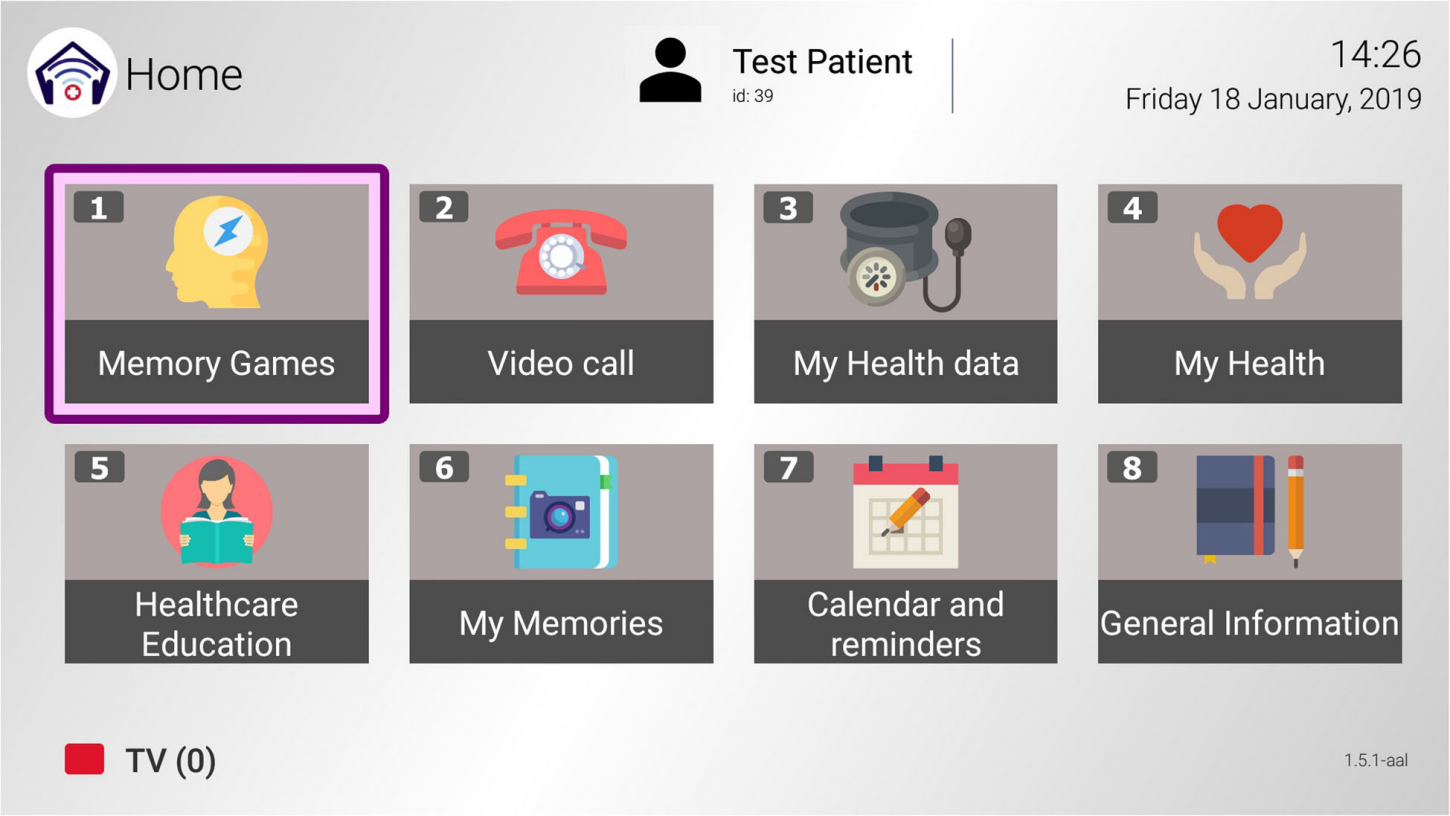
Main menu functionalities

This platform has three core services and three service-oriented applications (Fig. 2).

##### Core services

The set of core services is composed by general communication functionalities and other specific functionalities that will allow the system to collect user information.

- Calendar and reminders: Enables the dyad to schedule health related events such as daily health care or prescribed treatment (e.g. medication dose and time), ADLs (e.g. physical activities), celebrations (e.g. family birthdays and holidays) etc. On-screen messages appear to remind events while the patient watches the television.
- Health monitoring and data transmission to a health server: Records vital sign measurements such as blood pressure, pulse, glucose and weight and displays evolution in charts. This information will be automatically transmitted from the platform to a health server which researchers and caregivers will have access to.
- Videoconference: Allows bidirectional communication between patients and their caregivers or researches. Researches connect to the platform from a remote station for health monitoring and remote assistance and support, as well as distance health learning. Caregivers connect to the platform from a mobile device App or PC for social interaction.

##### Service-oriented applications

The set of service-oriented applications is composed by more specific functionalities that aim at stimulating cognition, accessing personal media and useful healthcare information.

- Cognitive stimulation: Includes specific software, Stimulus©, with a series of interactive exercises that train different cognitive processes [39]. These activities (+ 50) are classified around ten functional areas: attention, visual and auditory perception, working memory, long-term memory, calculation, reasoning, executive functions, visuomotor functions, language and processing speed. It is designed for tablet use but has been specifically adapted for TV-AssistDem.
- Reminiscences: Is a tool to create a personalized book of life, gathering memories of one’s self in the past using media (photos, videos and music).
- Healthcare education: Offers information and support resources on dementia and other chronic diseases for PMCI/MD and their caregivers: infographics, care guides and online videos.

Within one to two weeks after study inclusion and upon completion of the baseline assessments, patients assigned to the intervention group will be scheduled a TV-AssistDem installation and training visit. Following this visit, patients will receive weekly calls during the first month. The purpose of these calls is to encourage platform use and solve usage problems. A maximum of six calls will be made during the follow-up period depending on individual platform use.

#### Control group

Patients in the control group will receive treatment-as-usual and will also be assessed for 12 months. After the baseline visit in which all the variables of the study will be collected, follow-up visits will be carried out every 6 months.

#### Randomization

The randomization will be made using the block randomization technique. The blocks consist of 4 patients, two subjects per group [Intervention (I) and control (C)]: (IICC); (CCII); (CICI); (ICIC); (ICCI); (CIIC). The blocks will be marked with a number from 1 to 54 and they will be chosen at random to create the allocation sequence using a sequence of random numbers generated by the Microsoft Excel program with the function fx:RAND().

### Statistics

The flow of individuals will be shown schematically with counts and percentages in a CONSORT diagram. The analysis will be made following an intention-to-treat procedure. All variables collected will be summarized by group and by site at baseline and at final follow-up. Statistics considered for presentation for continuous measures in summary tables will be the mean, minima and maxima, and standard deviation, and if the criteria of normality are not met, the median and the first and third quartiles. Categorical variables will be summarized using counts and percentages. The baseline variables of the experimental group and the control group will be compared using the Chi-Square test for categorical variables and analysis of covariance for continuous variables.

For the primary outcome, a Linear Mixed Model analyses will be carried out as this approach is optimal to include subjects with missing data. First, an unadjusted analysis will be carried out and secondarily, in case that potentially confusing factors differ between the experimental group and the control group, despite the randomization, a supplementary multivariate analysis will be carried out. For secondary outcomes a Linear Mixed Model and Generalized Mixed Model analysis will be used for continuous and categorical variables respectively. Analysis will be two-tailed and alpha level set at 0.05.

#### Economic analysis

A cost-effectiveness and/or cost-utility analysis from a Health Service perspective (financer perspective) will be performed. The time horizon is 2 years and will include only direct health (medication and healthcare use of services including outpatient and emergencies visits and hospital admissions). The Quality-Adjusted Life Years (QALYs) will be calculated to estimate the benefit calculating the utility improvement measure by EuroQoL-5D-5 L.

#### User-behaviour analysis

Data on various platform usage variables will be collected for the intervention group. Regression analysis will be used to assess the relationship between the primary and secondary outcome variables and the usage variables in order to indicate which aspects of the platform use, most affect the outcomes. The results of the usability tests will be analysed using statistical methods to quantify the error rate, effectiveness and learning curve of the TV-AssistDem platform.

#### Missing data

Procedures can sometimes be considered when using statistical methods that fail in the presence of any missing values, or when in the case of multiple-predictor statistical models all the data for an individual would be omitted because of a missing value in one of the predictors. For analyses involving multiple regression analysis, a multiple imputation approach will be considered and used if statistically sound, depending on the proportion and pattern of missing values.

#### Methods to ensure validity and quality of data

Accurate and reliable data collection will be assured by verification and cross-check of the case report form (CRF) against the researcher’s records (source document verification). Source document verification will be conducted for 5% of data in subjects. A comprehensive validation check program utilizing front-end checks in the CRF will verify these data. Discrepancies and queries will be generated accordingly in the CRF for online resolution by the researcher at the site. In addition, the CRF data will be reviewed on an ongoing basis for medical and scientific plausibility.

#### Sample size

The main outcome measure is the total score of the QoL-AD, which ranges from 13 to 52. According to recent studies [40, 41], the standard deviation of the total score of the QoL-AD is 7.

For a minimum important clinical difference of 2.77 on the total QoL-AD score, with a standard deviation of 7, the effect size is 0.39. To compare two groups (Intervention and Control) using a two-sample, two-sided t-test with a 5% statistical significance level, the minimum number of evaluable patients required in each group is 100 (200 overall), to give a power of 80%. Given that it is a follow-up study, we must bear in mind that there will be losses along this one, therefore we will recalculate the sample size to take into account an expected percentage of losses of 20%. The number of subjects needed for each group will be 120 subjects (240 in total).

## Discussion

### Strengths

If the hypothesis is proven, these findings will demonstrate the potential improvement in quality of life in older adults with mild cognitive impairment or mild dementia when using TV-AssistDem. Numerous studies have developed technological solutions which facilitate remote support for PMCI/MD. What makes this study methodologically stand out is addressing QoL as a main outcome measure, using valid QoL scales, and its large sample size.

The uniqueness of this home TV-based intervention lies on its widespread accessibility and its integrative approach to QoL in PMCI/MD and their caregivers. Television sets have an extensive home penetration in older people exposed to the age-related digital divide, ensuring a broader implementation of TV-AssistDem than other ICTs. Moreover, this remote home-based assistive service has the potential to improve accessibility to health and support services for older people with limited access to standard services and health or mobility problems. In addition, TV-AssistDem offers a multi-modal intervention, which according to the literature, has proven to be more effective than single-focused ones. By combining technical, behavioral, educational and social support interventions, TV-AssistDem will provide significant support to PMCI/MD and relief to their caregivers.

### Challenges

There are several anticipated challenges: poor engagement to TV-AssistDem and connectivity problems. Patient engagement to the platform will be determinant. Three barriers must be taken into consideration: patient sensorial and/or psychomotor impairment, patient illiteracy, and patient negative attitudes and views towards ICTs. Even though television sets seem to be the most suitable for this population, sensorial and psychomotor impairments may difficult the use of the platform in some patients. Low literacy skills, though not so obvious a barrier, may prevent patients from fully engaging with TV-AssistDem. Moreover, while providing access and training on ICTs can help older adults cross the digital divide, negative attitudes and views towards these techs may limit TV-AssistDem use. Patients may disengage if they perceive the platform as too intrusive, invasive or time-consuming. Along with these engaging limitations, wireless or phone network connectivity problems may limit TV-AssistDem use on a daily basis. While little can be done to deal with this problem, researchers must face poor engagement by guaranteeing videoconferencing solutions tailored to specific needs. As the barriers and facilitators each dyad will face may differ, researchers will guide, support, and encourage each participating home.

### Limitations

Selection bias resulting from the missing data may be a potential limitation. Strategies such as the following will be carried out to diminish the latter: the sample size will be increased to 20% (expected losses), unreachable patients will be called three times in different days and hours and patients who do not attend the visits will be offered three additional appointments. A multiple imputation approach will be considered as dropout will be inevitable.

Researchers have received training on conducting the valid measures which will be used safely among older PMCI/MD and these assessments involve minimal risk. To ensure intervention standardization, it follows an exhaustive structured and professionals will be specifically trained. Furthermore, a manual for the researchers has been designed including: the working plan, the protocol, the different parts of the intervention and details to assess each variable. This will guarantee that the procedure can be replicated elsewhere.

### Future directions

Because of the upcoming demographic change in Europe, developing active and assistive living technological solutions, becomes necessary for the independent living of the elderly in the near future. Cost effective, accessible and multi-modal solutions which significantly impact daily life, family support and healthcare assistance in older adults, such as TV-AssitDem, will continue to rapidly advance to best approach diverse healthcare settings and different ageing population needs.

## Data Availability

The datasets will not be publicly available when the data is under analyzing but will become available in future publications. All access to data generated and/or analyzed during the current study will be limited to authorized personnel, the Investigator, and researchers. Authorized regulatory personnel have the right to inspect and copy all records pertinent to this study, but all efforts must be made to remove participants’ personal data.

## Abbreviations

ADLs: Activities of Daily Living
CRF: Case Report Form
CST: Cognitive Stimulation Therapy
EuroQoL-5D-5 L: European Quality of Life 5 Dimensions 5 Levels
GDS-15: Geriatric Depression Scale
IADL: Lawton Instrumental Activities of Daily Living Scale
ICTs: Information and Communications Technology
MMSE: Mini-Mental State Examination
PMCI/MD: People with mild cognitive impairment or mild dementia
QALYs: Quality-Adjusted Life Year
QoL: Quality of Life
QoL-AD: Quality of Life-Alzheimer’s Disease Scale
SUS: System Usability Scale
TV-AssistDem: TV-based Assistive Integrated Service for people with mild dementia or mild cognitive impairment
ZBI-12: Zarit Burden Interview

## References

[1] World Health Organization and Alzheimer’s Disease. Dementia: a public health priority. 2012.

[2] Calleo J, Stanley M (2008). Anxiety disorders in later life. Psychiatr Times.

[3] Panegyres PK, Berry R, Burchell J. Early Dementia Screening. Diagnostics (Basel, Switzerland) [Internet]. 21 de enero de 2016 [citado 25 de julio de 2018];6(1). Disponible en: http://www.ncbi.nlm.nih.gov/pubmed/26838803. 10.3390/diagnostics6010006 PMC4808821 26838803

[4] International Alzheimer’s Disease. The Global Impact of Dementia An analysis of prevalence, incidence, cost and trends. 2015.

[5] Bleijlevens MHC, Stolt M, Stephan A, Zabalegui A, Saks K, Sutcliffe C (2015). Changes in caregiver burden and health-related quality of life of informal caregivers of older people with dementia: evidence from the European RightTimePlaceCare prospective cohort study. J Adv Nurs.

[6] Johansson MM, Marcusson J, Wressle E (2015). Cognitive impairment and its consequences in everyday life: experiences of people with mild cognitive impairment or mild dementia and their relatives. Int Psychogeriatrics junio de.

[7] Fisher GG, Franks MM, Plassman BL, Brown SL, Potter GG, Llewellyn D (2011). Caring for individuals with dementia and CIND: findings from the aging, demographics, and memory study. J Am Geriatr Soc.

[8] Winblad B, Cedazo-Minguez A, Graff C, Johansson G, Jönsson L, Kivipelto M (2016). The lancet neurology commission defeating Alzheimer’s disease and other dementias: a priority for European science and society. Lancet Neurol.

[9] Cooper C, Mukadam N, Katona C, Lyketsos CG, Ames D, Rabins P (2012). Systematic review of the effectiveness of non-pharmacological interventions to improve quality of life of people with dementia. Int Psychogeriatr junio de.

[10] Kuyken W, Orley J, Power M, Herrman H, Schofield H, Murphy B (1995). The World Health Organization quality of life assessment (WHOQOL) - position paper from the World Health Organization. Soc Sci Med noviembre de.

[11] Khosravi P, Ghapanchi AH (2016). Investigating the effectiveness of technologies applied to assist seniors: a systematic literature review. Int J Med Inform junio de.

[12] Chinner A, Blane J, Lancaster C, Hinds C, Koychev I. Digital technologies for the assessment of cognition: a clinical review. Evid Based Ment Health [Internet]. 1 de mayo de 2018 [citado 25 de junio de 2018];21(2):67–71. Disponible en: http://www.ncbi.nlm.nih.gov/pubmed/29678927. 10.1136/eb-2018-102890 PMC10270380 29678927

[13] World Health Organization. ADHERENCE TO LONG-TERM THERAPIES: EVIDENCE FOR ACTION. WHO. World Health Organization; 2015.

[14] Arlt S, Lindner R, Rösler A, von Renteln-Kruse W. Adherence to medication in patients with dementia: predictors and strategies for improvement. Drugs Aging. 2008. 10.2165/0002512-200825120-00005 19021302

[15] Hudani ZK, Rojas-Fernandez CH (2016). A scoping review on medication adherence in older patients with cognitive impairment or dementia. Res Soc Adm Pharm noviembre de.

[16] Smith D, Janaka L, Weller C, Kennedy B, Winbolt M, Young C (2017). A systematic review of medication non- adherence in persons with dementia or cognitive impairment. PLoS One.

[17] NICE-SCIE. Dementia - Supporting people with dementia and their carers in health and social care. En London; 2006.

[18] Orrell M, Yates LA, Burns A, Russell I, Woods RT, Hoare Z, et al. Individual Cognitive Stimulation Therapy for dementia (iCST): study protocol for a randomized controlled trial. Trials. diciembre de 2012;13(1):172. 10.1186/1745-6215-13-172 PMC3522062 22998983

[19] Thorgrimsen L, Selwood A, Spector A, Royan L, de Madariaga LM, Woods RT (2003). Whose quality of life is it anyway?. Alzheimer Dis Assoc Disord.

[20] Logsdon RG, Gibbons LE, McCurry SM, Teri L (2002). Assessing quality of life in older adults with cognitive impairment. Psychosom Med mayo de.

[21] Logsdon RG, Gibbons LE, McCurry SM, Teri L (1999). Quality of life in Alzheimer’s disease: patient and caregiver reports. J Ment Heal Aging.

[22] Gómez-Gallego M, Gómez-Amor J, Gómez-García J (2012). Validación de la versión española de la escala QoL-AD en pacientes con enfermedad de Alzheimer, cuidadores y profesionales sanitarios. Neurologia..

[23] Devlin NJ, Krabbe PFM (2013). The development of new research methods for the valuation of EQ-5D-5L. Eur J Health Econ.

[24] Herdman M, Gudex C, Lloyd A, Janssen M, Kind P, Parkin D (2011). Development and preliminary testing of the new five-level version of EQ-5D (EQ-5D-5L). Qual Life Res diciembre de.

[25] Zarit SH, Reever KE, Bach-Peterson J (1980). Relatives of the impaired elderly: correlates of feelings of burden. Gerontologist diciembre de.

[26] Bedard M, Molloy DW, Squire L, Dubois S, Lever JA, O’Donnell M (2001). The Zarit burden interview: a new short version and screening version. Gerontologist..

[27] Haynes RB, Taylor DW, Sackett DL, Gibson ES, Bernholz CD, Mukherjee J (1980). Can simple clinical measurements detect patient noncompliance?. Hypertension..

[28] Sackett DavidL., Gibson EdwardS., Wayne Taylor D., Brian Haynes R., Hackett BrendaC., Roberts RobinS., Johnson ArnoldL. (1975). RANDOMISED CLINICAL TRIAL OF STRATEGIES FOR IMPROVING MEDICATION COMPLIANCE IN PRIMARY HYPERTENSION. The Lancet.

[29] Hansen RA, Kim MM, Song L, Tu W, Wu J, Murray MD (2009). Comparison of methods to assess medication adherence and classify nonadherence. Ann Pharmacother marzo de.

[30] Morisky DE, Green LW, Levine DM (1986). Concurrent and predictive validity of a self-reported measure of medication adherence. Med Care enero de.

[31] Val Jiménez A, Amorós Ballestero G, Martínez Visa P, Fernández Ferré ML, León Sanromà M. [Descriptive study of patient compliance in pharmacologic antihypertensive treatment and validation of the Morisky and Green test]. Aten primaria. octubre de 1992;10(5):767–770. 1472599

[32] Lawton MP, Brody EM (1969). Assessment of older people: self-maintaining and instrumental activities of daily living. Gerontologist enero de.

[33] Chisholm D, Knapp MR, Knudsen HC, Amaddeo F, Gaite L, van Wijngaarden B (2000). Client Socio-Demographic and Service Receipt Inventory-European Version: development of an instrument for international research. EPSILON Study 5. European Psychiatric Services: inputs Linked to Outcome Domains and Needs. Br J Psychiatry Suppl.

[34] Beecham J, Knapp M. 12 Costing psychiatric interventions. En: G. Thornicroft, editor. Measuring Mental Health Needs. 2^a^. 2001. p. 200–24.

[35] Tsoi Kelvin K.F., Chan Joyce Y.C., Hirai Hoyee W., Wong Adrian, Mok Vincent C.T., Lam Linda C.W., Kwok Timothy C.Y., Wong Samuel Y.S. (2017). Recall Tests Are Effective to Detect Mild Cognitive Impairment: A Systematic Review and Meta-analysis of 108 Diagnostic Studies. Journal of the American Medical Directors Association.

[36] Aguado C, Martínez J OM. Adaptación y validación al castellano de la versión abreviada de la “Geriatric Depresión Scale” (GDS) de Yesavage. Aten primaria / Soc Española Med Fam y Comunitaria. 2000;26 (supl 1:328.

[37] Brooke J (2013). SUS : a retrospective. J Usability Stud.

[38] Bangor A, Kortum P, Miller J (2009). Determining what individual SUS scores mean: adding an adjective rating scale. J Usability Stud.

[39] Stimulus [Internet]. (2018). citado 4 de julio de 2018.

[40] Goeman D, Comans T, Enticott JC, Renehan E, Beattie E, Kurrle S, et al. Evaluating the Efficacy of the “Support for Life” Program for People with Dementia and Their Families and Carers’ to Enable Them to Live Well: A Protocol for a Cluster Stepped Wedge Randomized Controlled Trial. Front Public Heal. 2016;4(November):245. 10.3389/fpubh.2016.00245 PMC5086896 27843893

[41] Woods RT, Orrell M, Bruce E, Edwards RT, Hoare Z, Hounsome B (2016). REMCARE: pragmatic multi-Centre randomised trial of reminiscence groups for people with dementia and their family carers: effectiveness and economic analysis. PLoS One.

